# Shortening of Subjective Visual Intervals Followed by Repetitive Stimulation

**DOI:** 10.1371/journal.pone.0028722

**Published:** 2011-12-16

**Authors:** Fuminori Ono, Shigeru Kitazawa

**Affiliations:** 1 Research Center of Advanced Science and Technology, The University of Tokyo, Tokyo, Japan; 2 Dynamic Brain Network Laboratory, Graduate School of Frontier Biosciences, Osaka University, Osaka, Japan; 3 Department of Brain Physiology, Graduate School of Medicine, Osaka University, Osaka, Japan; Université Pierre et Marie Curie, France

## Abstract

Our previous research demonstrated that repetitive tone stimulation shortened the perceived duration of the preceding auditory time interval. In this study, we examined whether repetitive visual stimulation influences the perception of preceding visual time intervals. Results showed that a time interval followed by a high-frequency visual flicker was perceived as shorter than that followed by a low-frequency visual flicker. The perceived duration decreased as the frequency of the visual flicker increased. The visual flicker presented in one hemifield shortened the apparent time interval in the other hemifield. A final experiment showed that repetitive tone stimulation also shortened the perceived duration of preceding visual time intervals. We concluded that visual flicker shortened the perceived duration of preceding visual time intervals in the same way as repetitive auditory stimulation shortened the subjective duration of preceding tones.

## Introduction

Substantial evidence indicates that the subjective experience of time does not exactly match the actual duration of events; rather, perceived durations stretch or contract depending on properties that are ostensibly independent of the elapsed time. For example, accumulated evidence shows that the perception of the duration of a time interval is lengthened by presenting the subject with repetitive tone stimuli [1], [2] or visual flickers [2]–[4] before the test interval. In fact, the durations of either a rapid series of tones [5]–[7] or flickering visual stimuli [8] are themselves perceived to be longer than the physical duration. To explain these changes in time perception, researchers have generally hypothesized that there is some kind of internal pacemaker [9] that is accelerated by repetitive stimulation. Acceleration of this pacemaker leads to an increase in pulse counts during a given period, which eventually leads to an increase in subjective duration. It is worth noting that these previous studies explained the effects of repetitive stimulation that was presented before or during a timed test interval.

The pacemaker framework is embedded in scalar expectancy theory (SET; [10]), which is one of the most popular contemporary models of time perception. SET proposes that temporal processing consists of three major components namely, a clock process (consisting of a pacemaker and an accumulator), a memory process (consisting of short-term- and reference-memory stores), and a comparator process (which aids decision making). A number of studies have attempted to manipulate the clock component of the model in either animals [11], [12] or humans [1], [3] and have provided evidence for a pacemaker–accumulator clock similar to that proposed by SET. However, much less attention has been paid to the memory and decision-making components of the model. One way to examine these components is to deliver an external perturbation during memory or decision processing and to evaluate its effects on time perception.

In our previous study [13], we examined whether repetitive tone stimuli (presented after a time interval) altered the perception of the preceding time interval in a postdictive manner. In the experiment, one trial consisted of reference and test intervals. The intervals were defined by delivering the first tone, followed by a silent period, followed by a second tone. Immediately after a test interval, a rapid series of tones (auditory flutter) was presented. The participants judged whether the test interval was longer than the reference interval. Interestingly, we found that the perceived duration of the preceding test interval was shortened by the occurrence of repetitive tone stimuli that followed the test interval (in what follows, this is referred to as a flutter effect). In addition, we showed that the flutter effect was not due to a framing effect. From our results, we proposed a postdictive evaluation mechanism that depends on the current rate of the internal pacemaker. Suppose, for example, that a person with a pacemaker of 10 pulses/s timed a 1-s interval that was immediately followed by a rapid series of tones. In this scenario, 10 pulses would be stored in his accumulator at the end of the 1-s interval; however, the succeeding series of tones would speed up the internal pacemaker to (for example) 11 pulses/s. We proposed that the stored pulse count (10 pulses) would be normalized by the clock speed (11 pulses/s) immediately after the end of pulse counting, in a postdictive manner. In this case, the perceived duration would be shortened by approximately 10% relative to when the clock speed remained constant (10 pulses/s).

In the present study, we examine whether the following repetitive stimulation effect is limited to auditory stimuli or whether it could also occur with visual stimuli. A fundamental question about time perception is whether the mechanisms underlying temporal judgments are universal and centralized in the brain or whether they are modality-specific and distributed [14]–[16]. Most psychophysical models of time perception have assumed the existence of an internal pacemaker that is common to both vision and audition [9], [17]–[22]. In contrast, some authors provided evidence that suggests that the timing of brief intervals is modality dependent [14], [23]–[25]. To examine this question, we presented a flickering visual stimulus after a test interval. If there is no such postdictive mechanism in the visual modality, the visual flicker stimulation should have no effect on subjective duration. If this postdictive mechanism is present in the visual system, the perceived duration of the visual interval should be shortened by the visual flicker.

## Experiment 1

In experiment 1, we examined the effect of a visual flicker that followed a test interval.

### Methods

#### Observers

Six paid volunteers participated; new participants were recruited for experiments 2–[Sec s4] [Sec s5] 5. All participants were unaware of the purpose of the experiment, had normal hearing, and had normal (or corrected-to-normal) visual acuity. The procedures were approved by the internal review board of Research Center for Advanced Science and Technology, The University of Tokyo, and written informed consent was obtained from all participants prior to the testing.

#### Apparatus and Stimuli

Participants were seated approximately 60 cm away from a monitor (Iiyama HM204D, 100 Hz) in a dark, quiet room. Experiments were run on a PC/AT compatible computer using a ViSaGe stimulus generator (Cambridge Research Systems). The visual markers were black filled or white filled geometric figures (a circle or a cross) presented in the center of the monitor on a gray background. The circle and the cross were 2° in diameter. We presented two kinds of stimuli (a circle and a cross) as visual markers to distinguish between the beginning (cross) and the end (circle) of each interval.

#### Procedure

**Figure 1:**
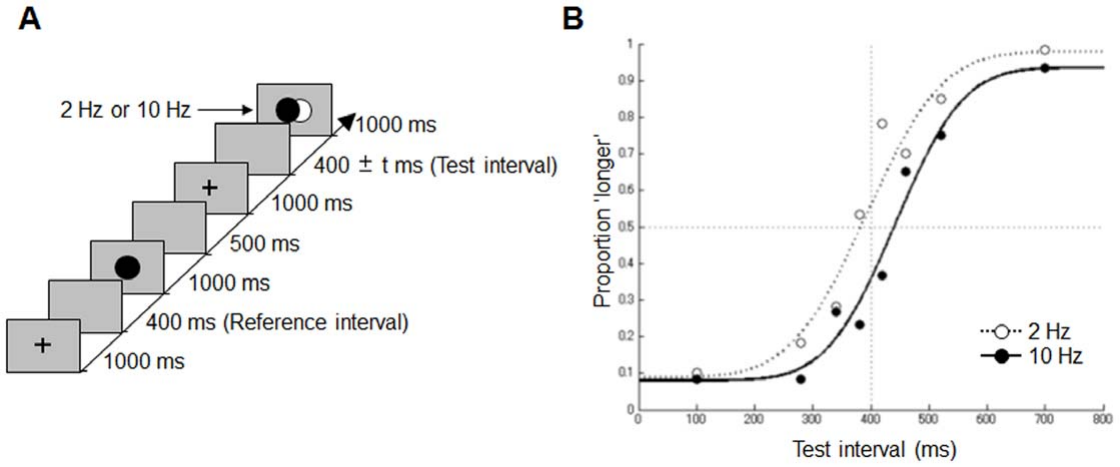
Trial sequence and results in Experiment 1. (a) The sequence of events in one trial is shown along a time-line. The overlapping black and white circles represent a visual flickering stimulus (2 Hz or 10 Hz). (b) The probability that the subjects judged the test interval to be longer than that of the reference interval is plotted against the test interval.

To examine the effect of a visual flicker that followed a test interval, we used the same type of task as in the previous studies [26], [27]. One trial consisted of a reference and a test interval (Figure 1A). Each trial was initiated by the participant by pressing the space bar. As soon as the space bar was pressed, a black cross was presented for 1000 ms, followed by a blank screen for 400 ms (reference interval), followed by a black circle being presented for 1000 ms. After a blank screen for 500 ms, a black cross was then presented for 1000 ms. This was followed by a blank screen for an interval that was chosen pseudorandomly from eight intervals (100, 280, 340, 380, 420, 460, 520, or 700 ms) (test interval); next, a circle was presented. The last circle flickered between black and white at either 2 or 10 Hz for 1000 ms. The participants were instructed to judge whether the test interval was longer than the reference interval and to respond by pressing a key that corresponded to each judgment. The interval to be estimated started from the offset of the first marker (cross) and ended at the onset of the second marker (circle). Each subject participated in 160 trials.

### Results and Discussion

The proportion of “longer” judgments was usually higher at 2 Hz than at 10 Hz (Figure 1B). The mean values of proportions of “longer” judgments were 0.55 (0.02) and 0.42 (0.01) at 2 and 10 Hz, respectively. Two-way ANOVAs with two within-subject variables revealed that the main effects of the flickering frequency (2 Hz or 10 Hz), the test interval (100, 280, 340, 380, 420, 460, 520, or 700 ms), and their interaction were significant (*F*(1, 5) = 12.64, *p* = .01, *F*(7, 35) = 44.82, *p*<.001, and *F*(7, 35) = 5.61, *p*<.001, respectively). Multiple comparisons (Ryan's method; [28]) showed that the subjective duration was significantly shorter at 10 Hz than at 2 Hz when the test interval was 380 and 420 ms (*p*<.05). The point of subjective equality, defined as the intersection of the cumulative Gaussian curve with the P = 0.5 line (that is, equal probability that the interval would be judged “longer” and “shorter”), was greater at 10 Hz (441 ms) than at 2 Hz (392 ms). When the data were analyzed subject-by-subject, the mean of the point of subjective equality was significantly larger at 10 Hz than at 2 Hz (paired t-test, *t*(5) = 2.66, *p* = .04). These results show that a time interval followed by a high-frequency visual flicker was perceived to be shorter than a time interval followed by a low-frequency visual flicker. These results also indicate that repetitive stimulation following the test interval results in an effect due to visual stimulation.

## Experiment 2

In experiment 1, the visual flicker followed each test interval. In experiment 2, we displayed the visual flicker before the test interval. According to previous studies ([2]–[4]), it is expected that a time interval preceded by a high-frequency repetitive stimulus will be perceived as longer than that preceded by a low-frequency repetitive stimulus.

### Method

**Figure 2:**
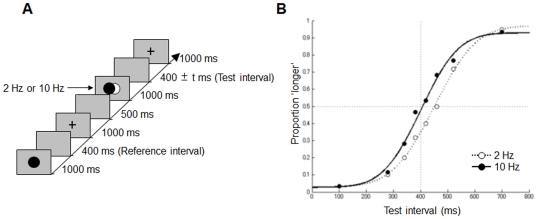
Trial sequence and results in Experiment 2. (a) The sequence of events in one trial is shown along a time-line. (b) The probability that the subjects judged the test interval to be longer than that of the reference interval is plotted against the test interval.

All aspects of this experiment were the same as those in the first experiment, except that the visual flicker was presented before the test interval (Figure 2A). Six paid volunteers participated; each participant completed 160 trials.

### Results and Discussion

The means of proportions of “longer” judgments were 0.40 (0.02) and 0.47 (0.02) at 2 and 10 Hz, respectively. Two-way ANOVAs revealed that the main effects of the flickering frequency (2 Hz or 10 Hz) and the test interval (*F*(1, 5) = 18.62, *p* = .007, and *F*(7, 35) = 73.88, *p*<.001, respectively) were both significant; however, their interaction was not significant (*F*(7, 35) = 0.66, *p* = .70) (Figure 2B). The point of subjective equality was smaller at 10 Hz (400 ms) than at 2 Hz (450 ms). When the data were analyzed subject-by-subject, the mean of the point of subjective equality was significantly smaller at 10 Hz than at 2 Hz (paired t-test, *t*(5) = 2.66, *p* = .04). It is worth noting that the effect of the same visual flickers was reversed, depending on whether the stimuli were presented before (experiment 2) or after (experiment 1) the test interval. Stimuli occurring before the test interval lengthened the subjective duration whereas those occurring after the test interval shortened the subjective duration.

## Experiment 3

The next question was whether the subjective time interval becomes shorter as the frequency of the succeeding visual flicker increases. In experiment 3, we examined whether the shortening effect due to a succeeding visual flicker generalizes over a range of 2–20 Hz.

### Method

**Figure 3:**
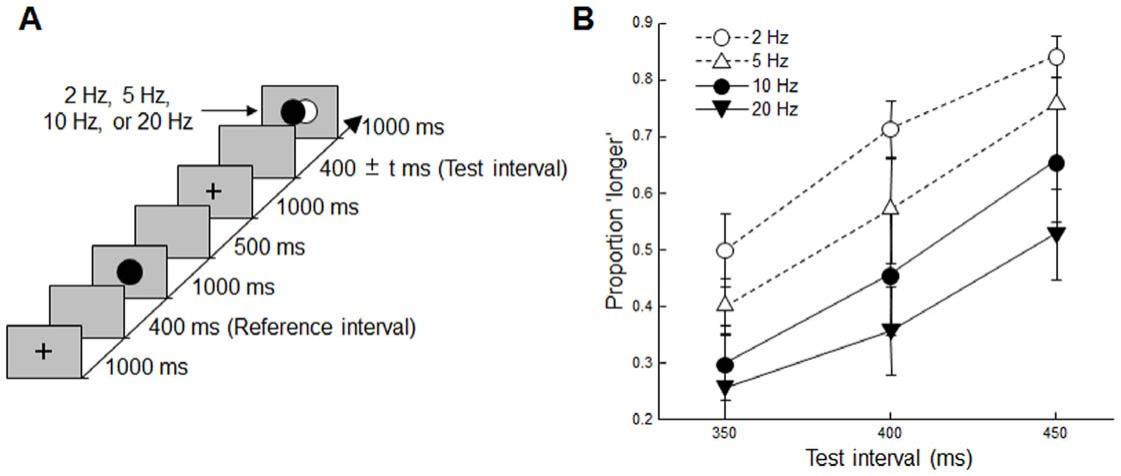
Trial sequence and results in Experiment 3. (a) The sequence of events in one trial is shown along a time-line. (b) The probability that the subjects judged the test interval to be longer than that of the reference interval is plotted against the test interval. Error bars show the standard error.

All aspects of this experiment were the same as those of experiment 1, except that the frequency of the succeeding series of visual flickers was chosen from four potential frequencies (2, 5, 10, or 20 Hz) and the test intervals were chosen from three different times (350, 400, or 450 ms) (Figure 3A). The range of 2–20 Hz was selected because a flicker stimulus appeared as a single continuous stimulus when the frequency was too high (flicker fusion). Seven paid volunteers participated, and each participant completed 120 trials.

### Results and Discussion

The means of proportion of “longer” judgments were 0.68 (0.03), 0.57 (0.06), 0.47 (0.08), and 0.38 (0.05) at 2, 5, 10, and 20 Hz, respectively. Two-way ANOVAs showed that the main effects of the flickering frequency (2, 5, 10, or 20 Hz) and the test interval (350, 400, or 450 ms) were both significant (*F*(3, 18) = 7.30, *p*<.002, and *F*(2, 12) = 19.04, *p*<.001, respectively); however, their interaction was not (*F*(6, 36) = 0.35, *p* = .90) (Figure 3B). Multiple comparisons showed that the subjective duration at 20 Hz was significantly shorter than that at either 2 Hz or 5 Hz and that the subjective duration at 10 Hz was significantly shorter than that at 2 Hz (*p*<.05). There was no significant difference between the nearby frequencies (2 Hz–5 Hz, 5 Hz–10 Hz, and 10 Hz–20 Hz). These results indicate that the subjective time interval became shorter as the frequency of the succeeding repetitive stimulation increased over the range of 2–20 Hz (see also Information, S1).

## Experiment 4

In experiments 1–[Sec s3] 3, the visual flickers were presented in the same position as the preceding test stimuli. The next question was whether the time perception would be affected by presenting the visual flickers that followed the test interval in a different location than in the preceding test stimuli. In experiment 4, we examined whether a flicker following the test interval in one hemifield of the visual field shortened the subjective preceding interval in the opposite hemifield.

### Method

**Figure 4:**
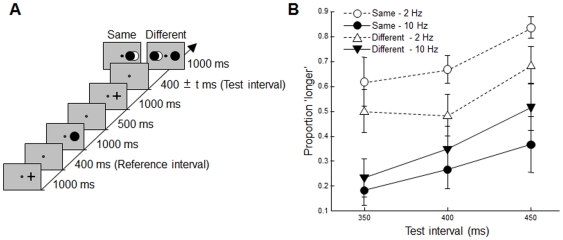
Trial sequence and results in Experiment 4. (a) The sequence of events in one trial is shown along a time-line. (b) The probability that the subjects judged the test interval to be longer than that of the reference interval is plotted against the test interval. Error bars show the standard error.

Stimuli were presented approximately 7.5° in diameter to the left and/or right side of the center of the display, a fixation dot (0.2° in diameter) was presented in the center of the display; the location of the stimuli was randomly changed in each trial (left, right, or left and right) (see Figure 4A). There were four conditions (same-2 Hz, same-10 Hz, different-2 Hz, and different-10 Hz). In the same-conditions (same-2 Hz and same-10 Hz), the flickering stimulus was presented on the same side as the first stimulus. In the different-conditions (different-2 Hz and different-10 Hz), the flickering stimulus was presented on a different side than the first stimulus, and the black circle was presented on the same side as the first stimulus. Thus, two stimuli were presented at the end of the trial in the different-conditions paradigm. We presented two visual stimuli in the different conditions so that participants were able to complete the task just by attending to one side of the visual field as in the same condition. The test intervals were chosen from three different times (350, 400, or 450 ms). Six paid volunteers participated; each participant completed 120 trials.

### Results and Discussion

The mean values of the proportion of “longer” judgments were 0.70 (0.05), 0.27 (0.06), 0.55 (0.06), and 0.36 (0.05) at same-2 Hz, same-10 Hz, different-2 Hz, and different-10 Hz conditions, respectively. Three-way ANOVAs showed that the main effects of the flickering frequency (2 Hz or 10 Hz) and the test interval (350, 400, or 450 ms) were both significant (*F*(1, 5) = 16.93, *p* = .009, and *F*(2, 10) = 4.78, *p* = .03, respectively); however, the flicker's location (same or different) was not significant (*F*(1, 5) = 0.25, *p* = .63). The interaction between the flicker's location and the flickering frequency was significant (*F*(1, 5) = 10.80, *p* = .02) (Figure 4B). Multiple comparisons showed that the subjective duration in same-10 Hz was significantly shorter than that in same-2 Hz and different-2 Hz. The subjective duration in different-10 Hz was significantly shorter than that in the same-2 Hz condition (*p*<.05). These results show that a time interval that is followed by a high-frequency visual flicker is perceived to be shorter than the one that is followed by a low-frequency visual flicker.

In experiment 4, we compared the subjective duration at 2 Hz and at 10 Hz in each hemifield because we were interested in the effect of the following flicker in the same or different hemifield. Planned contrast showed that the subjective duration in same-10 Hz was significantly shorter than that in same-2 Hz (*F*(1, 10) = 26.45, *p*<.001) and that the subjective duration in different-10 Hz was significantly shorter than that in different-2 Hz (*F*(1, 10) = 5.02, *p* = .04). These results show that the following flicker presented in one hemifield of the visual field shortens the subjective preceding interval in the opposite hemifield.

## Experiment 5

Experiments 1–[Sec s3] [Sec s4] 4 showed that, similar to the effect of auditory stimuli, visual stimuli influence the perception of the duration of preceding time intervals. This finding supports a “central timer” hypothesis, which suggests that there is a single timer for both visual and auditory modalities. However, it is still possible that there are two timers, one for each modality, and that each timer may only be affected by a stimulus of the same modality. This issue has been discussed before in articles on auditory and visual duration judgments [29]. Penton-Voak et al. (1996) [1] found that clicks affected the perception of the duration of both tones and visual stimuli. This suggests that click effects transfer between modalities, which may be stronger evidence for a central timer than obtaining an effect in only one modality. Therefore, in experiment 5, we examined whether the repetitive presentation of auditory stimuli shortened the perception of the duration of preceding visual time intervals.

### Method

**Figure 5:**
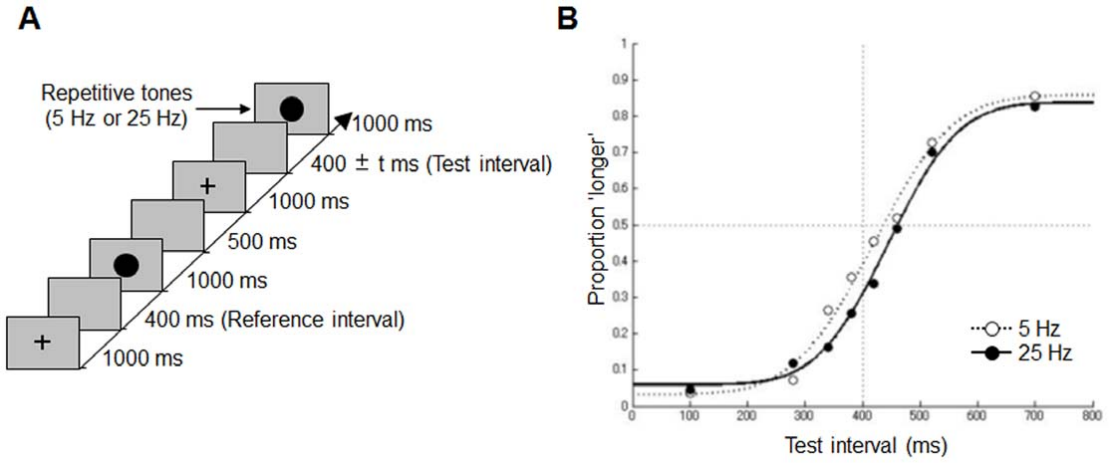
Trial sequence and results in Experiment 5. (a) The sequence of events in one trial is shown along a time-line. (b) The probability that the subjects judged the test interval to be longer than that of the reference interval is plotted against the test interval.

This method is similar to that of the experiment one; however, the auditory flutter sound was presented while the second marker of the target interval was presented, instead of the flickering visual stimuli (Figure 5A). The flutter stimulus was a tone burst (500 Hz) that lasted for 10 ms, including a rise time and a fall time of about 1 ms each. The tone bursts were repetitively presented at either 5 or 25 Hz for 1000 ms. The tone was presented to the ears at 70 dB SPL. Eleven paid volunteers participated; each participant completed 160 trials.

### Results and Discussion

The mean values of the proportion of “longer” judgments were 0.41 (0.02) and 0.37 (0.02) at 5 and 25 Hz, respectively. Two-way ANOVAs revealed that the main effects of the fluttering frequency (5 Hz or 25 Hz) and of the test interval were both significant (*F*(1, 10) = 5.11, *p* = .04, *F*(7, 70) = 44.07, *p*<.001, respectively); however, their interaction was not significant (*F*(7, 70) = 1.15, *p* = .34) (Figure 5B). The point of subjective equality was smaller at 25 Hz (418 ms) than at 2 Hz (445 ms). When the data were analyzed subject-by-subject, the mean of the point of subjective equality was significantly smaller at 10 Hz than at 2 Hz (paired t-test, *t*(10) = 2.71, *p* = .02). These results show that an auditory flutter that is presented immediately after the visual interval shortens the perception of the duration of the preceding visual interval. This suggests that the effects of repetitive stimulation following the test interval act across modalities. We infer that the repetitive presentation of visual stimuli would also shorten the perceived duration of preceding auditory intervals. We believe this speculation deserves to be confirmed in the future.

## Discussion

Our results show that a series of visual flickers presented after a test interval shortened the subjective duration of the preceding interval and that the effect depends on the frequency of the repetition. Specifically, the subjective duration shortened as the frequency of the visual flicker increased. In marked contrast, the subjective duration was lengthened when the visual flicker was delivered before the test interval. These findings indicate that the effect of repetitive stimulation after the test interval does occur with visual stimulation. In addition, it is worth noting that visual flickers presented after the test interval in one hemifield of the visual field shortened the subjective (preceding) interval in the opposite hemifield. Additionally, the auditory flutter presented just after the visual interval shortened the perceived duration of the preceding time interval.

The present findings have implications for our understanding of the mechanisms underlying temporal judgments in the brain. Previous research on time perception suggests theoretical explanations based on the assumption that there is a single, central timer in the brain [9], [17]–[22]. Our results support this single-timer hypothesis because repetitive simulation following the test interval had an effect on the auditory modality, the visual modalities, as well as the multimodal situation.

As noted in the introduction, the internal pacemaker theory [9], [22] predicts that subjective time intervals should become longer as the rate of repetitive presentation increases. In a clear departure from this prediction, our study showed that subjective time intervals became shorter as the frequency of repetitive stimuli increased. Thus, our results cannot be explained by the internal pacemaker theory *per se*; however, they do suggest that there are contributions from some other processes such as the memory or decision components in temporal processing. This agrees with theories that hypothesize that working memory forms the basis of temporal perception [30]–[34].

Recent studies [35], [36] have demonstrated that adaptation to a high-frequency visual stimuli decreased subjective duration of another visual stimulus in the location where the high-frequency stimulus had been presented. This phenomenon may seem analogous to the present finding. However, the present finding is quite different because the reduction of subjective duration occurred when the high-frequency stimuli were presented after the test interval, rather than before. In addition, the results of the fourth experiment suggest that repetitive stimulation after the test interval causes an effect that generalizes across the visual hemifield. These results exclude the possibility of explaining the present results in terms of adaptation to the high-frequency stimulus.

In summary, our findings are novel in two respects. First, the visual flicker shortened the preceding apparent time intervals in the same way as repetitive tone stimulation. Second, the effect generalizes across the visual field and across modality. These results have highlighted the robustness of postdictive modulation in time perception. In this study, we used repetitive visual flickers that differed in their frequency. However, we are able to manipulate other properties of visual stimuli, such as the size, shape, color, motion speed and complexity (e.g., facial expressions). The effects of these properties on the perception of the empty interval warrant further study in the future.

## Supporting Information

Information S1 **A preliminary experiment to examine the effect of non-repetitive stimulation.**
(DOC)
