# Supplementary material for: Shortening of Subjective Visual Intervals Followed by Repetitive Stimulation
Source: PLoS One. 2011 Dec 16;6(12):e28722. doi: 10.1371/journal.pone.0028722 (PMC3241676; doi:10.1371/journal.pone.0028722)
Supplement: Information S1 — A preliminary experiment to examine the effect of non-repetitive stimulation. (DOC) [file pone.0028722.s001.doc]

**S1. A preliminary experiment to examine the effect of non-repetitive stimulation**

We added an experiment to examine the effect of non-repetitive stimulation. Nine students participated in the experiment with the same procedure that was used in experiment 3, except that the frequency of repetitive stimuli was 0, 2, and 10 Hz. The means of proportion of ‘longer’ judgments were 0.45 (0.04), 0.38 (0.03), and 0.27 (0.02) at 0, 2, and 10 Hz, respectively. Paired t-tests showed that the subjective duration at 10 Hz was significantly shorter than 0 Hz (t(9) = 3.99, p = .003) and 2 Hz (t(9) = 3.41, p = .008). The results were consistent with the findings of the present study.
